# Analysis of gene expression in patients prior to TB treatment to identify those associated with pyrazinamide-hepatotoxicity

**DOI:** 10.5588/ijtldopen.25.0189

**Published:** 2025-08-13

**Authors:** N. Zielinski, T.T. Brehm, N. Köhler, P. Sánchez Carballo, D. Schaub, C. Lange, M. Reimann

**Affiliations:** ^1^ Research Center Borstel Leibniz Lung Center, Department of Clinical Infectious Diseases, Borstel, Germany;; ^2^ German Center for Infection Research, Partner Site Hamburg-Lübeck-Borstel-Riems, Borstel, Germany;; ^3^ University of Lübeck, Respiratory Medicine and International Health, Lübeck, Germany;; ^4^ University Medical Center Hamburg-Eppendorf, I. Department of Internal Medicine, Division of Infectious Diseases, Hamburg, Germany;; ^5^ Baylor College of Medicine and Texas Children’s Hospital, Global TB Program, Houston, United States;; ^6^ University Medical Center Hamburg-Eppendorf, Institute for Infection Research and Vaccine Development (IIRVD), Hamburg, Germany.

**Keywords:** tuberculosis, drug-susceptible TB, drug-induced liver injury

Dear Editor,

First-line drugs for the treatment of drug-susceptible TB (DS-TB) are the cornerstone of standard therapy, which consists of a highly-effective combination of ethambutol (EMB), isoniazid (INH), rifampicin (RIF) and pyrazinamide (PZA).^1^ However, hepatotoxicity due to DS-TB therapy occurs in 2–58% of treated patients, and can significantly impact their quality of life and interfere with treatment adherence.^2,3^ Treatment adjustments may be required, leading to drug interruption and prolonged treatment duration.^4^ Hepatotoxicity can present as an asymptomatic elevation in liver enzymes, bilirubin, alkaline phosphatase and gamma glutamyl transferase, with the potential to escalate to a state of life-threatening acute liver failure.^2^ PZA is associated with the highest risk of hepatotoxicity in first-line treatment, with reported incidence rates ranging from 5–58%.^5–7^ Risk factors include: female sex, age, history of alcohol abuse, chronic hepatitis-B- or -C-virus-infection and a genetic susceptibility.^2^ Although the mechanism of PZA-associated hepatotoxicity is poorly understood, it is likely to be as a result of PZA metabolic intermediates.^8^ As the mechanism is not yet understood, it would be highly desirable to identify patients at high risk of developing PZA-associated hepatotoxic adverse events prior to treatment initiation and to assess whether patient preconditions may increase the risk of hepatotoxic adverse events. We therefore aimed to identify genes that are differentially expressed in patients before the start of DS-TB treatment and associated with PZA-hepatotoxicity.

The present study used whole blood RNA data from adult patients with pulmonary DS-TB who were prospectively enrolled in three independent cohorts at the Research Center Borstel between 2013 to 2021. These observational cohorts were conducted to assess various clinical endpoints in patients with DS-TB and multidrug-resistant TB (MDR-TB). Clinical data were collected before, during and after treatment.^9^ The outcome in our study was defined as an increase in liver enzymes leading to discontinuation of PZA. The severity of the increased liver enzymes was retrospectively quantified using the Common Terminology Criteria for Adverse Events (CTCAE) grading scale (version 5.0). Significant differences in patient characteristics were identified using Wilcoxon rank sum test or Fisher's exact test. For the differentially expressed genes, Benjamini-Hochberg adjusted p-values were used. The significance level was set at a p-value <0.05 after adjustment for all analyses. Data preparation for the gene expression analysis is described elsewhere.^10^ Informed consent was obtained from all patients.

A total of 52 patients with pulmonary DS-TB on PZA-containing regimens were eligible for analysis. Out of those, 6 (11.5%) had to discontinue PZA due to hepatotoxicity. In 5 cases, PZA was the only first-line drug that was discontinued, and in one case, INH was also not reintroduced after treatment interruption. According to the CTCAE grading scale, 2 (33.3%) patients had a grade 2, 3 (50.0%) had a grade 3 and 1 (16.6%) had a grade 4 hepatotoxic adverse event. Hepatotoxicity occurred at a median of 22 days (interquartile range [IQR] 11–43 days) after starting TB treatment. Female patients had a significantly higher risk of PZA-associated hepatotoxicity (odds ratio: 10.4, 95% confidence interval: 1.2–135.8). No significant differences were found in TB disease and treatment-related factors (including PZA dosage), comorbidities (including hepatitis B, hepatitis C), behavioral aspects (including alcohol consumption), baseline liver enzyme levels, or other sociodemographic characteristics (including age). Before the start of treatment, 237 out of 44,000 transcripts were statistically significantly differentially expressed when comparing patients with and without PZA-associated hepatotoxicity (Supplementary Data Table S1). The three most upregulated gene transcripts, as indicated by their log fold change (logFC), are *BCL2L11* (logFC: 1.3), *ESRG* (logFC: 1.2) and *TNXB* (logFC: 1.1). In contrast, the three most downregulated gene transcripts are *IFIT2* (logFC: -1.2), *HMGB2* (logFC: -1.1) and *PRNP* (logFC: -1.1). Z-score normalized gene expression of all 237 transcripts is hierarchically mapped in the Figure. The heat map can be separated into two main clusters. Cluster 1 includes 42 patients, with 1 patient developing PZA-associated hepatotoxicity (2.4%). Cluster 2 seems to be at higher risk as 5 out of 10 patients (50.0%) experienced hepatotoxicity during the course of PZA treatment. For the patient in cluster 1, hepatotoxicity manifested only after 62 days of PZA treatment, a much later occurrence than in all other patients. When comparing the patient characteristics between the clusters, female patients were significantly more likely to be in cluster 2 (14.3% vs. 50.0%; p-value = 0.025) but no other significant differences were found.

**Figure.**
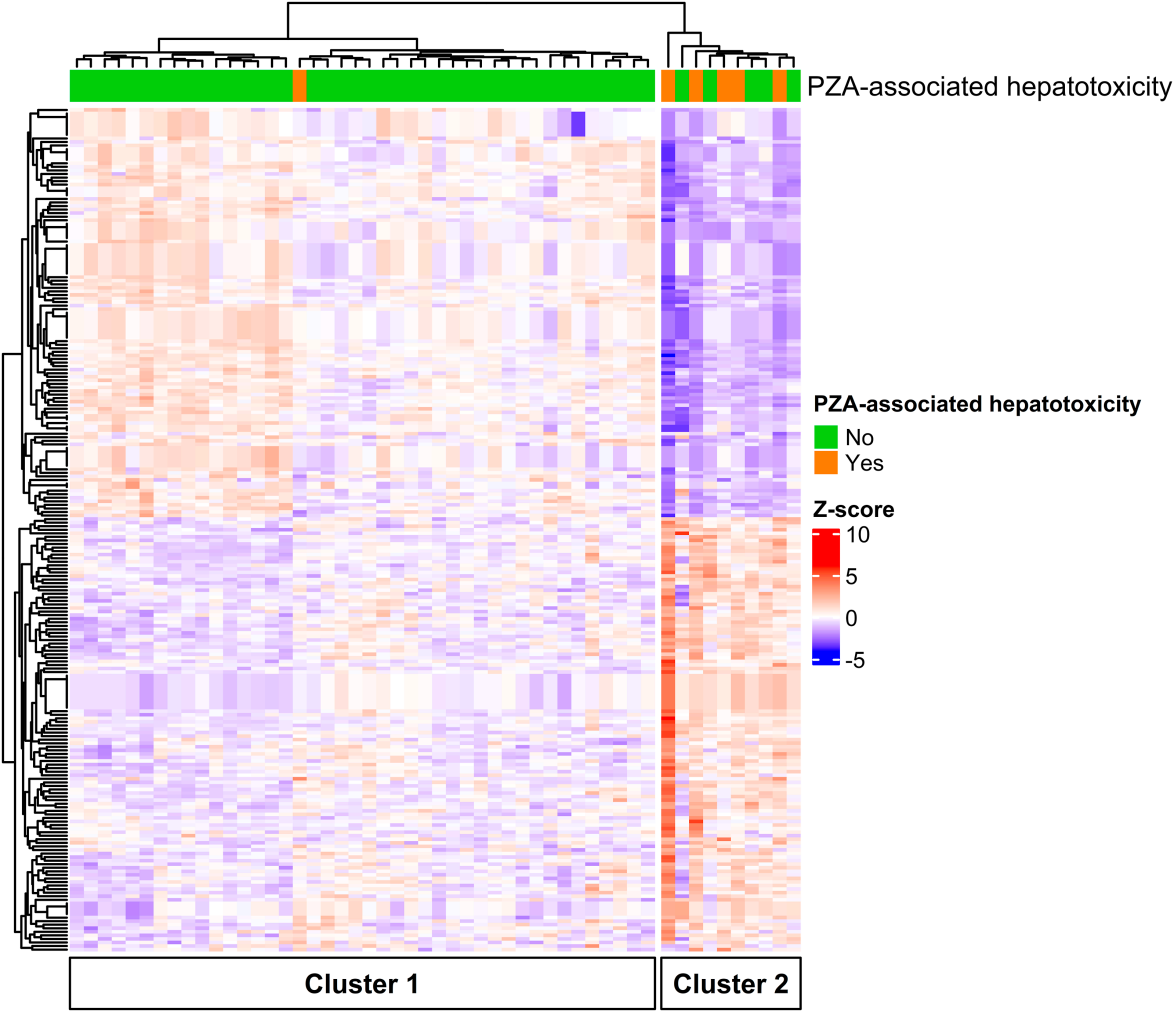
Heat map of 237 (z-score normalized) significantly differentially expressed transcripts. PZA = pyrazinamide

Among the 237 statistically significantly differentially expressed transcripts, one gene (*BACH1*) stands out due to its previously reported association with anti-TB drug-induced liver injury (AT-DILI).^11^ Findings from two studies in Japanese and Chinese cohorts suggest that *BACH1* polymorphisms may be associated with increased susceptibility to AT-DILI, potentially mediated through impaired regulation of oxidative stress pathways, although the exact molecular mechanisms remain unclear.^12,13^ Although our study did not investigate genetic polymorphisms, we observed a statistically significantly downregulation of *BACH1* (logFC: -1.02) in patients with PZA-associated hepatotoxicity. Notably, previous studies focused on the entire first-line DS-TB regimen rather than on PZA specifically. With regard to PZA-associated hepatotoxicity, previous studies have focused on differential gene expression and metabolism during PZA treatment in animal models.^14,15^ However, none of the genes identified in those studies were differentially expressed in our dataset, likely reflecting the absence of PZA exposure at the time of analysis in our cohort.

Our study presents novel findings, as it demonstrates that differential gene expression occurs in patients prior to the administration of PZA. A strength of the study is that the data is based on closely monitored and well characterized DS-TB patients. Based on the distinct clustering, there may be potential to use pre-treatment transcriptomic data as a risk stratification or prognostic tool to predict PZA-associated hepatotoxicity. However, a larger sample size is required to facilitate further evaluation of this finding.

Future studies should investigate the potential of identifying a transcriptome-based biomarker for predicting hepatotoxicity.

## Supplementary Material


