# Supplementary material for: Analysis of gene expression in patients prior to TB treatment to identify those associated with pyrazinamide-hepatotoxicity
Source: IJTLD Open. 2025 Aug 13;2(8):497–9. doi: 10.5588/ijtldopen.25.0189 (PMC12352952; doi:10.5588/ijtldopen.25.0189)
Supplement: Supplementary file 1 [file ijtldopen25-0189_supplementarydata1.pdf]

Supplementary Table 1. Statistically significantly differentially expressed transcripts (n=237).

A_33_P3229527	*BTNL8*	*HNRNPH3*	*MAP7*	*RFK*
A_33_P3231677	*C1orf52*	*HNRNPH3*	*MECP2*	*RHOB*
A_33_P3250323	*C6orf106*	*HNRNPH3*	*MICU2*	*RNASET2*
A_33_P3265866	*C6orf120*	*HNRNPR*	*MORF4L1*	*RNF138*
A_33_P3291160	*C8G*	*HSD17B12*	*MRPL18*	*RNF19A*
A_33_P3318766	*CA5B*	*IAPP*	*MSANTD4*	*SBNO1*
A_33_P3324552	*CBX3*	*IDS*	*MSANTD4*	*SCRN3*
A_33_P3332749	*CCAR1*	*IFIT2*	*MSANTD4*	*SEP15*
A_33_P3360007	*CCAR1*	*IGSF9*	*MSANTD4*	*SERINC1*
A_33_P3381292	*CCAR1*	*IMPAD1*	*MSANTD4*	*SNX19*
A_33_P3402086	*CCAR1*	*IP6K2*	*MSANTD4*	*SNX2*
ACTR3	*CCAR1*	*IRF8*	*MSANTD4*	*SPEM1*
ACTR3	*CCAR1*	*JUND*	*MTO1*	*SPG11*
ACTR3	*CCAR1*	*KATNA1*	*MTRF1*	*SRSF2*
ACTR3	*CCAR1*	*KDM4C*	*NADK2*	*SSX3*
ADCY7	*CCAR1*	*KHDRBS1*	*NAV2-AS5*	*TBC1D22A*
ADSS	*CCDC124*	*KIAA1551*	*NCK1*	*TDG*
AF289570	*CCDC157*	*KRAS*	*NDFIP1*	*TET1*
AK090442	*CENPE*	*KRTAP3-2*	*NDNF*	*TEX13B*
AKT2	*CMPK1*	*LDHA*	*NDUFA6-AS1*	*THC2569478*
ANAPC4	*CNST*	*LINC00273*	*NEMF*	*THC2661718*
AOC2	*CYCS*	*LINC00689*	*NYNRIN*	*THC2708197*
AP5S1	*DDX6*	*lnc-ACOT1-3*	*OTUD5*	*TLE3*
APOBEC3G	*DHX36*	*lnc-GLP2R-1*	*PAFAH1B2*	*TNXB*
ARPC3	*DUX4*	*lnc-KIAA0825-1*	*PCNP*	*TNXB*
ATP2B4	*EFHC2*	*lnc-TMC7-1*	*PDE4DIP*	*TP53TG3*
AUH	*ENST00000390428*	*LOC100129129*	*PHF8*	*TRIM37*
B2M	*ENST00000439431*	*LOC100130540*	*PI4K2B*	*TTYH1*
B2M	*ENST00000510538*	*LOC101928038*	*PIKFYVE*	*TXLNG*
B2M	*ENST00000560295*	*LOC101928837*	*PKI55*	*UBLCP1*
B2M	*ENST00000562306*	*LOC143286*	*PLEKHF2*	*UNKL*
B2M	*ERMN*	*LOC153811*	*PNPT1*	*UNKL*
BACH1	*ERMN*	*LOC441528*	*PPP2R3C*	*UNKL*
BCL2L11	*ESRG*	*LOC641746*	*PRDM2*	*UNKL*
BLOC1S5	*ETNK1*	*LOC728061*	*PRNP*	*USO1*
BRAF	*EVA1C*	*LOC728673*	*PRNP*	*UTP3*
BRAF	*FAM134A*	*LOC93444*	*PRNP*	*VAMP7*
BRAF	*FAM208B*	*LRRTM1*	*PRNP*	*VEGFA*
BRAF	*FUZ*	*LSM14A*	*PRNP*	*VPS54*
BRAF	*FZD4*	*LTV1*	*PRNP*	*VWA2*
BRAF	*GAPVD1*	*LTV1*	*PRNP*	*WDR76*
BRAF	*GATC*	*LTV1*	*PRNP*	*YTHDF2*
BRAF	*GNG13*	*LTV1*	*PSMA1*	*YTHDF2*
BRAF	*GOLT1B*	*LTV1*	*RAD50*	*ZNF714*
BRAF	*GSAP*	*LTV1*	*RAD50*	*ZNF768*
BRD3	*HELQ*	*LY6G5B*	*RB1*	
BRD7	*HMGB2*	*LY6G6D*	*RDH10*	
BTBD1	*HNRNPA2B1*	*MAFG-AS1*	*RETNLB*	
